# Effectiveness and Durability of mRNA Vaccine-Induced SARS-CoV-2-Specific Humoral and Cellular Immunity in Severe Asthma Patients on Biological Therapy

**DOI:** 10.3389/fimmu.2022.892277

**Published:** 2022-05-20

**Authors:** Michal Podrazil, Pavla Taborska, Dmitry Stakheev, Michal Rataj, Jan Lastovicka, Alena Vlachova, Petr Pohunek, Jirina Bartunkova, Daniel Smrz

**Affiliations:** ^1^ Department of Immunology, Second Faculty of Medicine, Charles University, and Motol University Hospital, Prague, Czechia; ^2^ Department of Pneumology, Second Faculty of Medicine, Charles University, and Motol University Hospital, Prague, Czechia; ^3^ Department of Pediatrics, Second Faculty of Medicine, Charles University, and Motol University Hospital, Prague, Czechia

**Keywords:** severe asthma patients on biological therapy, SARS-CoV-2, COVID-19 vaccination, cellular immunity, humoral immunity

## Abstract

Coronavirus disease 2019 (COVID-19) vaccines effectively elicit humoral and cellular immunity against severe acute respiratory syndrome coronavirus 2 (SARS-CoV-2) in healthy populations. This immunity decreases several months after vaccination. However, the efficacy of vaccine-induced immunity and its durability in patients with severe asthma on biological therapy are unknown. In this study, we evaluated the effectiveness and durability of mRNA vaccine-induced SARS-CoV-2-specific humoral and cellular immunity in severe asthma patients on biological therapy. The study included 34 patients with severe asthma treated with anti-IgE (omalizumab, n=17), anti-IL5 (mepolizumab, n=13; reslizumab, n=3), or anti-IL5R (benralizumab, n=1) biological therapy. All patients were vaccinated with two doses of the BNT162b2 mRNA vaccine with a 6-week interval between the doses. We found that this COVID-19 vaccination regimen elicited SARS-CoV-2-specific humoral and cellular immunity, which had significantly declined 6 months after receipt of the second dose of the vaccine. The type of biological treatment did not affect vaccine-elicited immunity. However, patient age negatively impacted the vaccine-induced humoral response. On the other hand, no such age-related impact on vaccine-elicited cellular immunity was observed. Our findings show that treatment of patients with severe asthma with biological therapy does not compromise the effectiveness or durability of COVID-19 vaccine-induced immunity.

## Introduction

Severe acute respiratory syndrome coronavirus 2 (SARS-CoV-2) is the cause of the COVID-19 pandemic, and the efforts to fight this disease largely rely on prophylactic vaccination (1). The currently used COVID-19 vaccines provide a high level of protection against SARS-CoV-2 infection and severe forms of COVID-19 (2–6). These COVID-19 vaccines induce strong humoral and cellular responses in healthy populations (7). The humoral response leads to the production of anti-SARS-CoV-2 antibodies, which prevent the virus from infecting host cells and spreading in the body (8, 9). The cellular response, which largely relies on cytotoxic and memory T cells, prevents the virus from replicating in infected cells and protects patients from severe forms of COVID-19 (10–13). Despite the high performance of the current COVID-19 vaccines in eliciting robust protective immunity, the durability of this immunity, both humoral and cellular, has been found to be much shorter than previously expected (14–16). This finding led to a change in the vaccination strategy and administration of booster doses to reinvigorate antiviral immunity (17).

Unlike healthy populations, patients with many diseases have shown decreased efficacy for COVID-19 vaccines in eliciting protective immunity. This has been shown, for instance, in recipients of solid-organ transplants (18, 19), cancer patients (20), patients on dialysis (21), and patients receiving certain drugs (22). In addition to the compromised immunogenicity of current vaccines in these patients, the durability of the elicited immunity is still largely unknown.

Patients with severe asthma on biological treatment were initially expected to be at higher risk of SARS-CoV-2 infection and severe COVID-19 (23, 24). More recent studies, however, have not supported this expectation (25–27). Currently, there is no definite consensus on whether patients on these treatment plans have higher risks of SARS-CoV-2 infection and severe disease than the healthy population, and therefore, an individualized approach is recommended for these patients (28).

A recent study showed that vaccination of patients with severe asthma on biological therapy with the BNT162b2 mRNA vaccine was safe and tolerated, with a minimal impact on the control of their primary disease (29). However, the immunogenicity of this vaccine in these patients has not been evaluated.

In the present study, we evaluated the immunogenicity of the BNT162b2 mRNA vaccine and the durability of vaccine-elicited immunity in a cohort of 34 severe asthma patients on biological therapy, which is already a centralized standard of care in the Czech Republic. This cohort included patients on anti-IgE (omalizumab), anti-IL5 (mepolizumab, reslizumab), or anti-IL5R (benralizumab) therapy (30, 31). The immunogenicity of the vaccine was evaluated by assessing specific humoral and cellular responses after the administration of the first and second doses of the vaccine. The persistence of the elicited immunity was assessed six months after the administration of the second dose of the vaccine.

## Materials and Methods

### Patients and COVID-19 Vaccination

The inclusion criteria for patients for this study were no reported history of COVID-19 and no previously reported positivity for SARS-CoV-2. The study enrolled 37 patients with severe asthma who were on biological therapy and previously planned to undergo voluntary vaccination against COVID-19 with the BNT162b2 mRNA vaccine (Comirnaty, Pfizer-BioNTech, Mainz, Germany). Severe asthma patients on biological therapy underwent clinical and laboratory monitoring at regular three-month intervals at specialized allergy and pneumology centers of the Motol University Hospital in Prague. Blood samples were obtained between April 2021 and November 2021. The planned COVID-19 vaccination regimen included 2 doses of the BNT162b2 mRNA vaccine administered at a 6-week interval. According to the ongoing treatment regimen with monoclonal antibodies for severe asthma, a 48-h interval between COVID-19 vaccination and biologic administration was used. The patient samples for analysis included peripheral blood serum and unclotted peripheral blood and were collected during the planned health check-ups. A complete blood count with differential was performed. The serum and peripheral blood mononuclear cells (PBMCs) were isolated and cryopreserved as previously described (32, 33). Adverse reactions to mRNA vaccination were monitored. Each patient provided signed written informed consent for the use of their blood-derived products for future research. The study was approved according to the ethical standards of the institutional research committee – the Ethics Committee of the Motol University Hospital in Prague (EK-346/21) – and performed in compliance with the 1964 Helsinki Declaration and its later amendments or comparable ethical standards.

### Determination of the Serum Levels of Anti-SARS-CoV-2 Antibodies

Patient sera were analyzed for the presence of anti-SARS-CoV-2 antibodies. Anti-SARS-CoV-2-spike glycoprotein receptor-binding domain (RBD) IgA and IgG antibodies were evaluated using IVD EIA COVID-19 RBD IgA or IgG (TestLine Clinical Diagnostics, Brno, Czech Republic). Anti-SARS-CoV-2 nucleocapsid protein (NCP) IgG antibodies were assessed using CLIA COVID-19 NP IgG (TestLine Clinical Diagnostics). The analyses were performed according to the manufacturer’s instructions. The levels of the specific antibodies are expressed according to the manufacturer’s instructions (in U/ml). The samples with U/ml values below 18 were considered negative, and levels above 18 U/ml were considered positive. Samples with antibody levels above the dynamic range of the method were reanalyzed using adequately diluted serum.

### Enrichment of Patient PBMCs With SARS-CoV-2-Reactive T Cells

Cryopreserved PBMCs were processed as previously described with minor changes (33). Briefly, cells were reconstituted (2 × 10^6^ cells/ml) overnight in human plasma serum-containing medium and then stimulated with overlapping peptides (0.5 µg/ml) of the SARS-CoV-2 spike glycoprotein (PepMix™ SARS-CoV-2 Spike Glycoprotein, cat.# PM-WCPV-S-1, JPT Peptide Technologies, Berlin, Germany). The stimulated cells were then cultured (37°C, 5% CO_2_) for 8 days in the presence of IL-2 (PeproTech, Cranbury, NJ) to enrich the peptide-reactive T cells.

### SARS-CoV-2 Spike Glycoprotein Peptide Reactivity of Enriched PBMCs

Eight-day-enriched PBMCs were restimulated with SARS-CoV-2 spike glycoprotein-derived peptides (0.5 μg/ml) and cultured for 5 h in the presence of brefeldin A (BioLegend, San Diego, CA) added 1 h after stimulation was initiated. Samples stimulated with vehicle alone (the peptide solvent) were used as a control. After the stimulation, the cells were stained with a fixable live/dead stain, fixed, permeabilized, and stained with fluorophore-labelled CD3-, CD4-, CD8-, IFN-γ-, and TNF-α-specific antibodies as described previously (33). The stained cells were analyzed by flow cytometry (FACSAria II, Becton Dickinson, Heidelberg, Germany). FlowJo software (Tree Star, Ashland, OR) was used to analyze the acquired flow cytometry data. The percentages of the peptide-reactive cytokine-producing T-cell populations were calculated as the difference in the percentage of cytokine-producing T cells between the vehicle- and peptide-stimulated samples.

### Statistical Analysis

Values were calculated from the indicated sample size (*n*) using GraphPad Prism 6 (GraphPad Software, La Jolla, CA). The Wilcoxon matched-pair signed-rank test was used to calculate the statistical significance (^NS^ *p>*0.05, *p<0.05, **p<0.01, ***p<0.001, ****p<0.0001) of differences between two groups. Matched-pair one-way ANOVA (Friedman test) with Dunn’s posttest or one-way ANOVA (Kruskal–Wallis test) with Dunn’s posttest was used to determine the statistical significance (^NS^ *p>*0.05, *p<0.05, **p<0.01, ***p<0.001, ****p<0.0001) of differences among three or more groups. Spearman’s rank-order correlation coefficient (*r*) and the correlation’s statistical significance (*p*) were used to determine the associations between two variables. A *p* value below 0.05 was considered significant. Biorender.com was used to produce graphical images (accessed in January 2022, license number XM23WFJ35Q).

## Results

### COVID-19 Vaccination Induces High Levels of Anti-SARS-CoV-2 Spike Glycoprotein Antibodies, Which Are Significantly Decreased Six Months After the Administration of the Second Dose of the Vaccine

**Figure 1:**
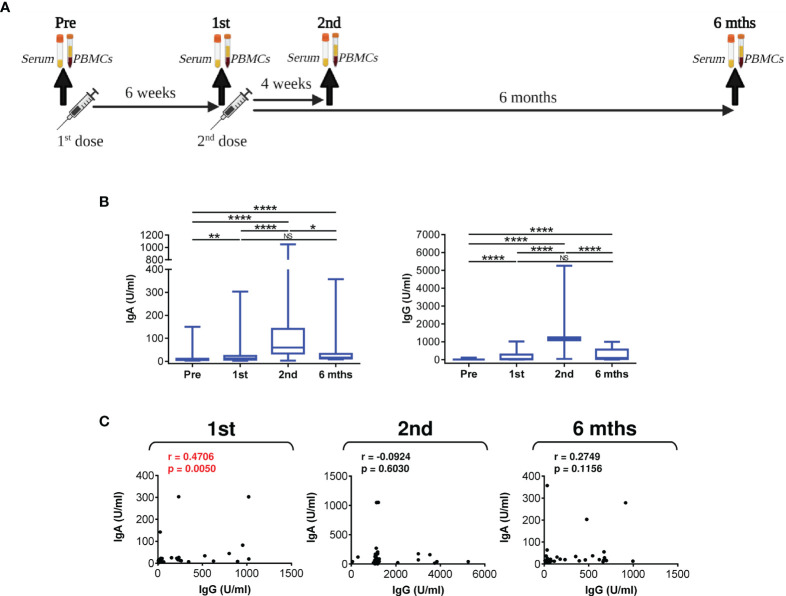
The serum levels of anti-RBD IgA and IgG antibodies during and after vaccination. **(A)** The sample collection strategy. **(B)** Serum levels (U/ml) of anti-RBD IgA (*left panel*) and IgG (*right panel*) antibodies before the first (Pre) and second (1st) vaccine doses were administered and 4 weeks (2nd) and 6 months (6 mths) after the second vaccine dose was administered. **(C)** Correlations between the serum levels (U/ml) of anti-RBD IgA and IgG antibodies determined in **(B)** In B, box and whisker plots (2.5–97.5 percentile) are shown, and the significance of differences among the groups (Pre, 1st, 2nd, and 6 mths) is indicated (NSp>0.05, *p<0.05, **p<0.01, ****p<0.0001; *n* = 34; matched-pair one-way ANOVA with Dunn’s posttest). In C, Spearman’s rank-order correlation coefficient (r) and the significance (*p* value; *n* = 34) are indicated.

Thirty-seven patients with severe asthma were enrolled in the study. The inclusion criteria were no previous history of COVID-19 or positive tests for SARS-CoV-2 and ongoing management of the patient’s primary disease by biological therapy indicated according to GINA recommendations (34, 35). The cohort of 37 enrolled patients comprised 18 patients on omalizumab (anti-IgE therapy), 14 patients on mepolizumab (anti-IL5), 4 patients on reslizumab (anti-IL5), and 1 patient on benralizumab (anti-IL5R) therapy. The patients’ median age was 57 years (range 21–73 years), and the cohort included 22 women and 15 men. Patient baseline characteristics collected before the first COVID-19 vaccine dose are shown in **Table 1** and further detailed in **Table S1**. All patients were administered two doses of the SARS-CoV-2 spike glycoprotein-based mRNA vaccine BNT162b2 with a 6-week interval between the two doses. We maintained a minimum interval of 48 h between COVID-19 vaccination and the administration of biologics. Samples were obtained within 1 week before the administration of the first and second doses of the vaccine and then 4 weeks and 6 months after the administration of the second dose of the vaccine (**Figure 1A**). Eighteen (49%) patients were completely free of any reactions. Nineteen (51%) patients reported commonly described side effects, most of which were classified as very common/common side effects and occurred after the second dose was administered. No differences were reported according to the ongoing biological therapy (data not shown).

**Table 1:** The cohort characteristics.

All	Patients (no.)	Patients(%)	Age(median, range)	Race	
**All**	**37**	**100**	**57, 21–73**	**Caucasian**	
Women	22	59.5	55, 21**–**71	Caucasian	
Men	15	40.5	57, 21**–**73	Caucasian
	**All**	**Omalizumab (anti-IgE)**	**Mepolizumab** **(anti-IL5)**	**Reslizumab** **(anti-IL5)**	**Benralizumab** **(anti-IL5R)**
**Patients** (no.)	**37**	18	14	4	1
**Treatment** (months) (median, range)\	**34, 1–146**	73, 17–146	20, 1–44	33, 30–34	9
**BMI** (median, range)	**26, 17–41**	25, 17**–**35	27, 20–41	26, 21–30	27
**Blood eosinophils** (cells/µl) (median, range)	**110, 0–990**	165, 30–990	45, 20–290	135, 50–210	0
**Total IgE** (IU/ml) (median, range)	**231, 17–3067**	254, 17–799	132, 20–3067	173, 39–293	606
**ECP** (ng/ml) (median, range)	**12, 3–150**	12, 3–150	12, 4–68	18, 8–28	10
**FeNO** (ppb) (median, range)	**43, 5–232**	36, 5–232	64, 6–158	86, 35–114	43

We first evaluated whether the 37 enrolled patients had been infected with SARS-CoV-2 before or during the study to eliminate interference with vaccine performance. The marker of a previous SARS-CoV-2 infection is the presence of anti-SARS-CoV-2 nucleocapsid protein (NCP) IgG antibodies in the serum (36). We found that before vaccination and 6 months after vaccination, 34 patients were negative (33 patients) or only borderline (1 patient) for anti-NCP IgG antibodies (**Figure S1A**). Among these 34 patients, only two patients had elevated prevaccination levels of anti-SARS-CoV-2 spike glycoprotein receptor-binding domain (RBD) IgA antibodies, and one patient had elevated prevaccination levels of anti-RBD IgG antibodies (**Figure S1B**). However, since these patients were negative for anti-NCP IgG antibodies and prepandemic antibodies raised against human seasonal coronaviruses were reported to cross-react with SARS-CoV-2 antigens (37), these patients were still included in the analyses. The patient with a borderline anti-NCP IgG antibody titer was also included, and this patient was, in contrast, found to be negative for prevaccination anti-RBD IgA and IgG antibodies (**Figure S1C**). The remaining 3 patients of the 37 enrolled patients had prevaccination levels of anti-RBD IgA and IgG antibodies that were also considered negative (**Figure S1C**). However, these 3 patients had elevated prevaccination levels of anti-NCP IgG antibodies, which were then decreased 6 months after vaccination, indicating a distant prevaccination SARS-CoV-2 infection in the past (36) (**Figure S1A**). Therefore, to minimize the impact of possible SARS-CoV-2 infection on the evaluation of COVID-19 vaccine performance in these patients during the study, we excluded these 3 patients from further analyses.

To determine COVID-19 vaccine performance in eliciting a humoral response in the tested patients, we analyzed the serum levels of anti-RBD IgA and IgG antibodies during and after vaccination. As shown in **Figure 1B**, the serum levels of anti-RBD IgA and IgG antibodies were significantly increased after the administration of the first dose of the vaccine. These serum levels were further and significantly increased after the administration of the second dose of the vaccine (**Figure 1B**). However, 6 months after the second dose of the vaccine was administered, these levels had returned to the levels observed after the administration of the first dose (**Figure 1B**). As shown in **Figure 1C**, the serum levels of anti-RBD IgA and IgG antibodies were found to correlate after the first dose of the vaccine was administered but not 4 weeks or 6 months after the second dose of the vaccine was administered. This finding indicated that the second dose of the vaccine promoted IgG antibody production more than IgA antibody production (**Figure 1C**). Collectively, the data show that the immunogenicity of the BNT162b2 mRNA vaccine is sufficient to induce strong humoral immunity in severe asthma patients on biological therapy but that this immunity then significantly declines within 6 months of the administration of the second dose of the vaccine.

### COVID-19 Vaccine Induces SARS-CoV-2 Spike Glycoprotein-Specific CD4^+^ and CD8^+^ T-Cell Immunity, Which Is Significantly Decreased Six Months After the Administration of the Second Dose of the Vaccine

**Figure 2:**
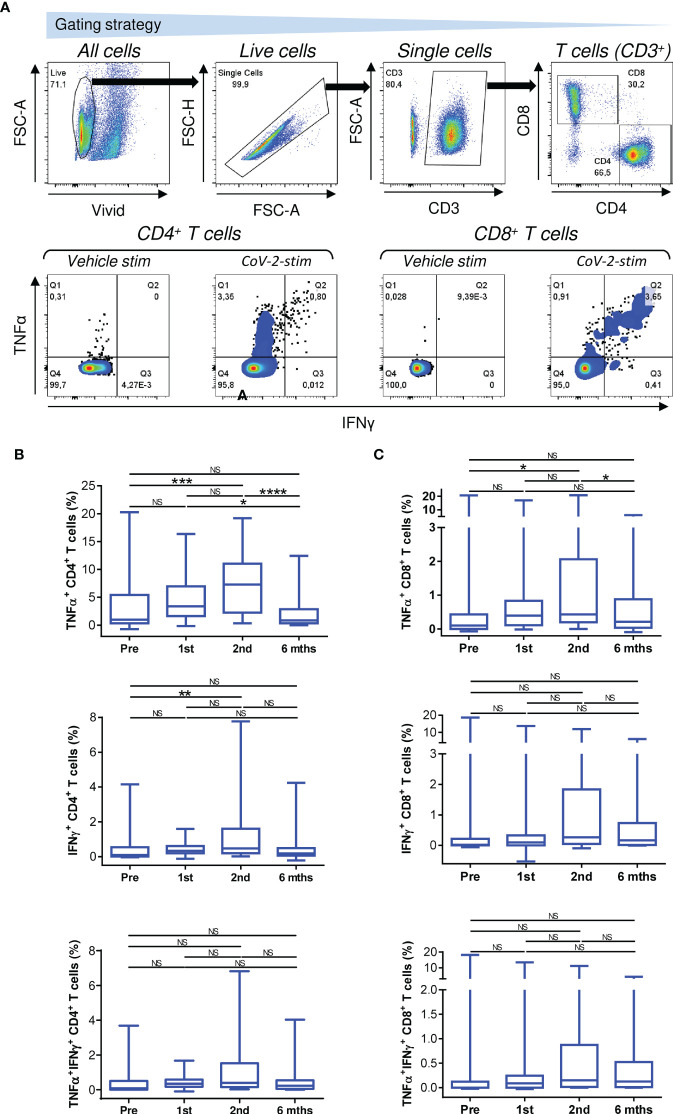
Reactivity of enriched PBMCs to SARS-CoV-2 spike glycoprotein peptides. **(A)** The gating strategy for flow cytometry data (*top panels*). The frequencies of TNFα-, IFNγ-, or TNFα/IFNγ-producing CD4^+^ and CD8^+^ T cells in enriched PBMCs stimulated with vehicle (Vehicle-stim) or SARS-CoV-2 spike glycoprotein-derived peptides (CoV-2-stim) (*bottom panel*s) determined by intracellular cytokine staining. The frequency of each peptide-reactive cytokine-producing T-cell population was calculated as the difference in the frequency of the cytokine-producing T cells between the vehicle- and peptide-stimulated samples. **(B, C)** The frequencies of peptide-reactive TNFα-, IFNγ-, or TNFα/IFNγ-producing CD4^+^ **(B)** and CD8^+^ **(C)** T cells before the first (Pre) and second (1st) vaccine doses were administered and 4 weeks (2nd) and 6 months (6 mths) after the second vaccine dose was administered. In B–C, box and whisker plots (2.5–97.5 percentile) are shown, and the significance of differences among the groups (Pre, 1st, 2nd, and 6 mths) is indicated (^NS^ *p > *0.05, *p < 0.05, **p < 0.01, ***p < 0.001, ****p < 0.0001; *n* = 34; matched-pair one-way ANOVA with Dunn’s posttest).

Cellular immunity against SARS-CoV-2 is important for efficient protection against the virus (38). Unlike humoral immunity, which is mediated by antibodies that immediately neutralize the virus, cellular immunity can only mobilize against a virus after antigen challenge (39–41). This mobilization relies on both CD4^+^ T cells and CD8^+^ T cells recognizing appropriate target antigens and effectively proliferating after antigen recognition. To exclude the possibility that the biological therapies used to treat the patients compromised both of these abilities of T cells, we tested the cellular immunogenicity of the vaccine using peptides derived from the SARS-CoV-2 spike glycoprotein (33, 42). These peptides were used to enrich patient PBMCs in peptide-reactive TNFα-, IFNγ- or TNFα/IFNγ-producing CD4^+^ or CD8^+^ T-cell populations *in vitro* (**Figure 2A**).

We found that COVID-19 vaccination enhanced the mobilization of SARS-CoV-2-reactive TNFα-producing CD4^+^ and CD8^+^ T cells, and this enhancement was significant after the second dose of the vaccine was administered (**Figures 2B, C**, top panels). However, this enhanced mobilization had significantly declined to prevaccination levels 6 months later (**Figures 2B, C**, top panels).

**Figure 3:**
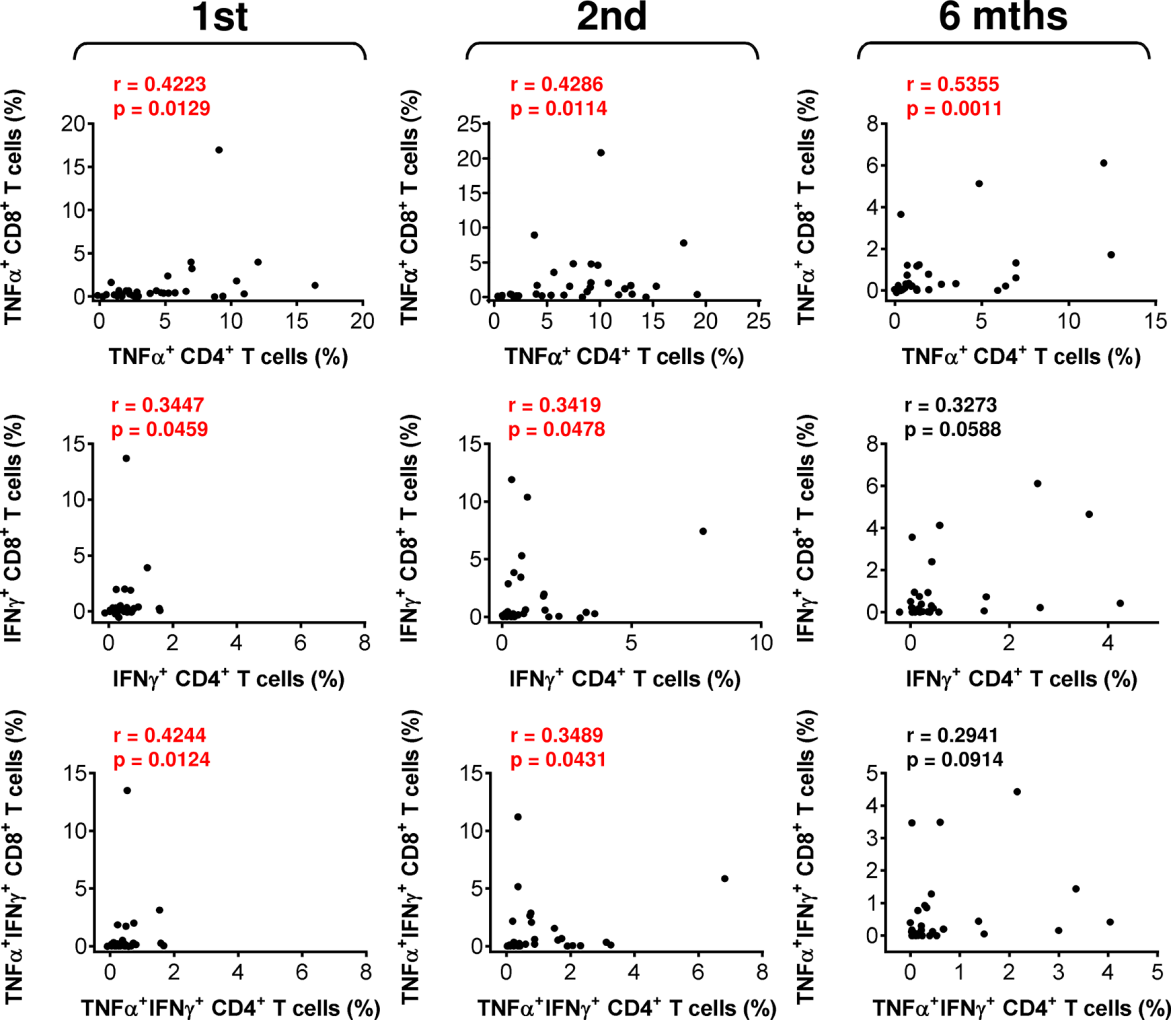
The associations between SARS-CoV-2 spike glycoprotein peptide-specific CD4^+^ and CD8^+^ T-cell reactivity during and after vaccination. The correlations between the frequencies of peptide-reactive TNFα-, IFNγ-, or TNFα/IFNγ-producing CD4^+^ and CD8^+^ T cells before (1st) and 4 weeks (2nd) and 6 months (6 mths) after the second vaccine dose was administered. Spearman’s rank-order correlation coefficient (r) and the significance (*p* value; *n* = 34) are indicated.

The mobilization of SARS-CoV-2-reactive IFNγ- or TNFα/IFNγ-producing CD4^+^ or CD8^+^ T-cell populations showed a tendency similar to that of TNFα-producing T cells (**Figures 2B, C**, middle and bottom panels), but the cohort data reached significance for only IFNγ-producing CD4^+^ T cells after the administration of the second dose of the vaccine (**Figure 2B**, middle panel). However, the data did show that the cellular mobilization of reactive cytokine-producing CD4^+^ and CD8^+^ T cells was significantly correlated (**Figure 3**), indicating polyfunctional responses by patient SARS-CoV-2-reactive T cells after antigen challenge. These data show that the immunogenicity of the BNT162b2 mRNA vaccine is sufficient to significantly promote cellular immunity in severe asthma patients on biological therapy but that this immunity, similar to humoral immunity, significantly declines within 6 months of the administration of the second dose of the vaccine.

### COVID-19 Vaccine-Induced Humoral Immunity Correlates With SARS-CoV-2-Specific CD8^+^ T-Cell Immunity

**Figure 4:**
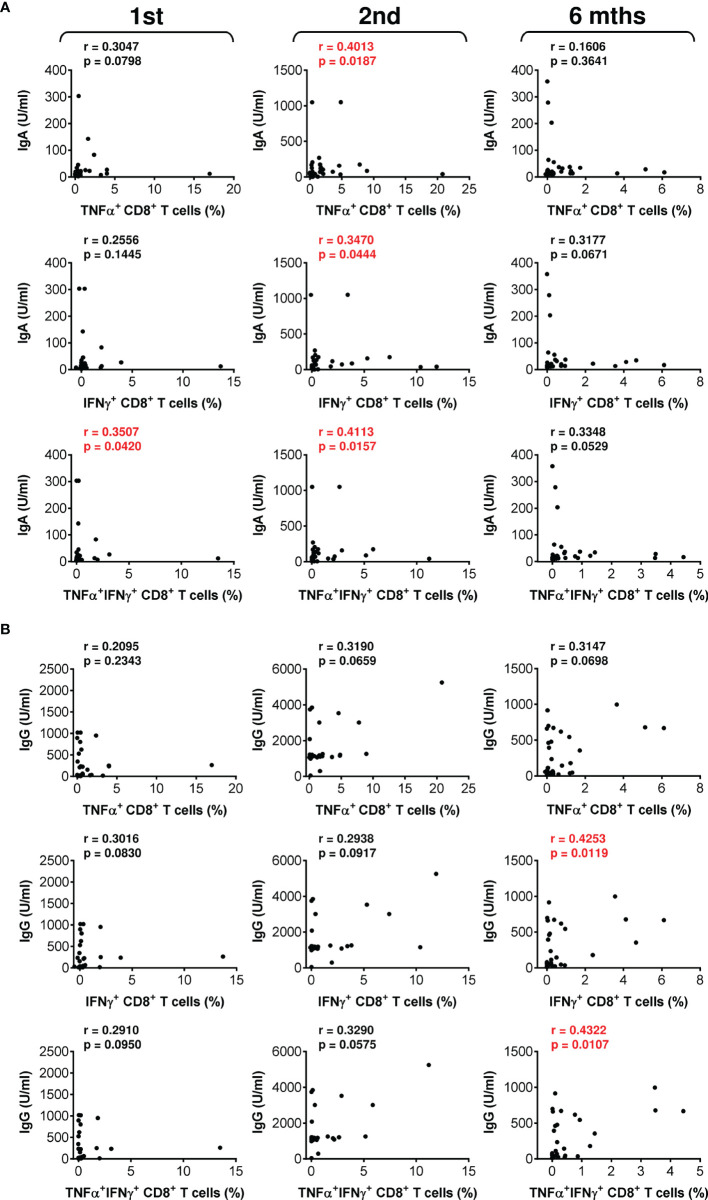
The associations between SARS-CoV-2 spike glycoprotein peptide-reactive CD8^+^ T cells and the serum levels of anti-RBD antibodies during and after vaccination. **(A, B)** The correlations between the frequencies of peptide-reactive TNFα-, IFNγ-, or TNFα/IFNγ-producing CD8^+^ T cells and serum levels (U/ml) of anti-RBD IgA **(A)** or IgG **(B)** antibodies before (1st) and 4 weeks (2nd) and 6 months (6 mths) after the second vaccine dose was administered. Spearman’s rank-order correlation coefficient (r) and the significance (*p* value; *n* = 34) are indicated.

The data showed that the COVID-19 vaccine induced both humoral and cellular immunity in severe asthma patients on biological therapy. Next, we analyzed whether there was a correlation between the extents of vaccine-elicited humoral and cellular immunity. We found that the serum levels of anti-RBD IgA and IgG antibodies minimally correlated with SARS-CoV-2-reactive CD4^+^ T cells (**Figure S2**). On the other hand, the correlations between antibody serum levels and SARS-CoV-2-reactive CD8^+^ T cells were more apparent (**Figure 4**). These findings showed that COVID-19-induced SARS-CoV-2-specific humoral immunity is more proportional to the CD8^+^ T-cell compartment.

### Immunogenicity of the COVID-19 Vaccine Is Comparable Among Severe Asthma Patients on Different Biological Therapies

The COVID-19 vaccine effectively induced SARS-CoV-2-specific immunity in the studied cohort of 34 patients on biological therapy. We next analyzed whether the type of biological therapy impacted vaccine performance. We stratified the patients into 3 groups based on therapy type. The first group included 17 patients on anti-IgE therapy (omalizumab), the second group included 13 patients on anti-IL5 therapy (mepolizumab), and the third group included 3 patients on anti-IL5 therapy (reslizumab). One patient on a different drug, an anti-IL5R therapy (benralizumab), was excluded from these comparisons. Among the 3 groups, there were no significant differences in patient age or total IgE or eosinophil cationic protein (ECP) serum levels (**Figure S3**). The only difference in the tested parameters among the groups was the eosinophil count in the peripheral blood, which differed between the group treated with anti-IgE therapy (omalizumab) and the group treated with anti-IL5 therapy (mepolizumab) (**Figure S3**).

**Figure 5:**
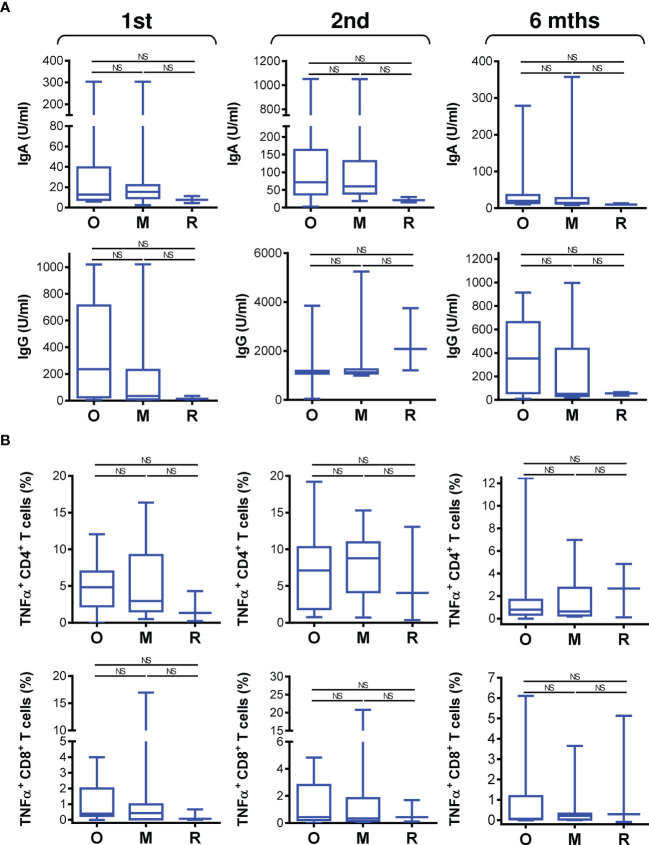
The impact of biological therapy on SARS-CoV-2 spike glycoprotein-specific humoral and cellular immunity during and after vaccination. **(A)** Patients were stratified into 3 groups based on the type of biological therapy they received. The groups were 17 patients on anti-IgE therapy (O; omalizumab), 13 patients on anti-IL5 therapy (M, mepolizumab), and 3 patients on anti-IL5 (R, reslizumab) therapy. The serum levels (U/ml) of anti-RBD IgA (*top panels*) and IgG (*bottom panels*) antibodies before (1st) and 4 weeks (2nd) and 6 months (6 mths) after the second vaccine dose was administered. **(B)** The frequencies of peptide-reactive TNFα-producing CD4^+^ (*top panels*) and CD8^+^ (*bottom panels*) T cells in the stratified groups in A before (1st) and 4 weeks (2nd) and 6 months (6 mths) after the second vaccine dose was administered. In **(A, B)**, box and whisker plots (2.5–97.5 percentile) are shown, and the significance of differences among the groups (O, M, and R) is indicated (^NS^ *p > *0.05; *n* = 17 (O), 13 (M), or 3 (R); one-way ANOVA with Dunn’s posttest).

We found that the type of biological therapy had no significant impact on COVID-19 vaccine-elicited humoral immunity (**Figure 5A**). There was also no impact on vaccine-elicited cellular immunity (**Figure 5B**). These findings revealed that vaccine performance was similar in all patients regardless of the type of biologic received.

### COVID-19 Vaccine-induced Humoral but Not Cellular Immunity Is Negatively Affected by the Age of Severe Asthma Patients on Biological Therapy

Thus far, we had not identified a clinical parameter that negatively affected COVID-19 vaccine performance in the studied cohort of severe asthma patients. We next analyzed whether any of the other clinical parameters evaluated affected vaccine-elicited humoral or cellular immunity. We found that the total IgE and ECP serum levels did not correlate with vaccine-elicited humoral (**Figure S4**) or cellular immunity (**Figures S5, S6**). Additionally, the peripheral blood eosinophil count did not correlate with vaccine-elicited CD8^+^ T-cell immunity (**Figure S7**). However, the eosinophil count correlated with the serum levels of vaccine-elicited anti-RBD IgG antibodies after the administration of the first dose and 6 months after the administration of the second dose of the vaccine (**Figure S8A**). After the administration of the first vaccine dose, the eosinophil count also correlated with vaccine-elicited TNFα- or IFNγ-producing CD4^+^ T cells (**Figure S8B**, top and middle panels).

**Figure 6:**
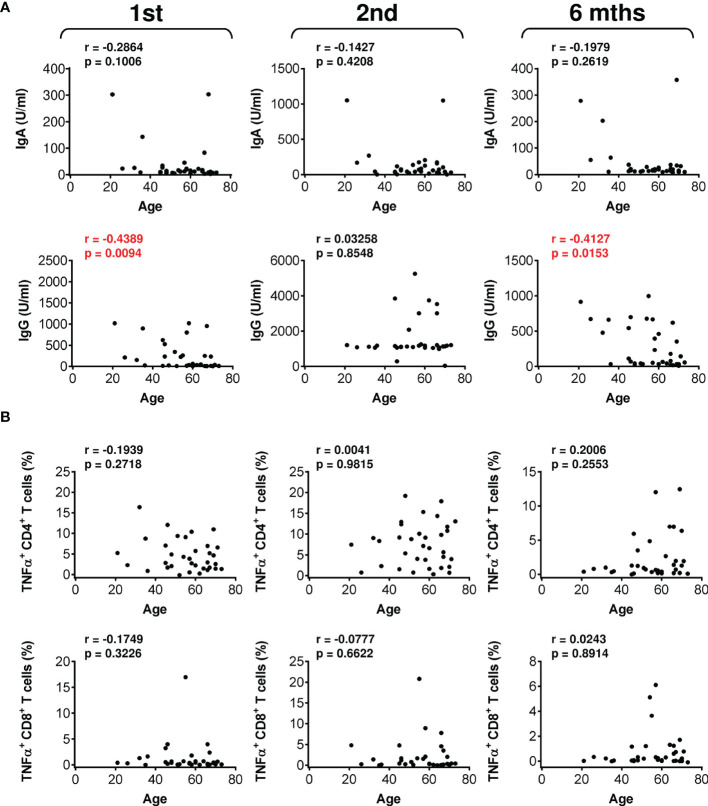
The impact of patient age on the serum levels of anti-RBD antibodies and SARS-CoV-2 spike glycoprotein peptide-reactive CD4^+^ and CD8^+^ T cells during and after vaccination. **(A)** The correlations between patient age and the serum levels (U/ml) of anti-RBD IgA (*top panels*) and IgG (*bottom panels*) antibodies before (1st) and 4 weeks (2nd) and 6 months (6 mths) after the second vaccine dose was administered. **(B)** The correlations between patient age and the frequencies of peptide-reactive TNFα-producing CD4^+^ (*top panels*) and CD8^+^ (*bottom panels*) T cells before (1st) and 4 weeks (2nd) and 6 months (6 mths) after the second vaccine dose was administered. In **(A, B)**, Spearman’s rank-order correlation coefficient (r) and the significance (*p* value; *n* = 34) are indicated.

We next analyzed whether patient age could be a clinical parameter impacting vaccine performance. Indeed, analyses revealed that patient age negatively affected the serum levels of vaccine-elicited anti-RBD IgG antibodies after the administration of the first dose and 6 months after the administration of the second dose of the vaccine (**Figure 6A**). In contrast, patient age did not negatively impact vaccine-elicited cellular immunity (**Figure 6B** and **Figure S9**). These data showed that vaccine-elicited humoral immunity was more sensitive to patient age than was cellular immunity.

## Discussion

This study showed that the COVID-19 vaccine used could elicit both humoral and cellular immunity against SARS-CoV-2 in patients with severe asthma on biological therapy. The type of biological therapy had no impact on either type of vaccine-elicited immunity. However, similar to the observed trend in healthy populations, vaccine-elicited immunity was significantly decreased 6 months after vaccination.

Biological therapy is increasingly used in clinical practice to treat severe asthma patients (43). Apart from treating the disease, it impacts the immune system. Patients with severe asthma are often treated with biologics, which are strong immunomodulators. Omalizumab binds to free IgE antibodies, which lowers free IgE levels and causes downregulation of FcϵRI receptors on basophils and mast cells (30). IgE antibodies are also important for cross-talk between innate and adaptive immunity and play roles in the susceptibility to respiratory infections (44) and in antiviral responses (45). As such, anti-IgE monoclonal antibodies were suggested as a potential treatment for COVID-19 that could enhance antiviral responses to the virus (46). Mepolizumab and reslizumab (anti-IL5 therapy) bind to IL-5 and thereby stop IL-5 from binding to its receptor on the surface of eosinophils. Inhibiting IL-5 binding to eosinophils reduces blood, tissue, and sputum eosinophil levels. Benralizumab specifically binds to IL-5Rα, thereby preventing the interaction between IL-5 and its receptor. Simultaneously, benralizumab, through its Fc region, binds to the receptor FcγIIIRa expressed by natural killer cells, thus inducing eosinophil apoptosis through the release of proapoptotic proteins such as granzymes and perforins (31). However, even though anti-IL5 therapy decreases the eosinophil count, this decrease does not prevent activation of any remaining eosinophils and subsequent humoral and cellular responses to viral challenge (47). Similar results were observed with anti-IL5R therapy, where the risk of respiratory infections due to a decreased eosinophil count was not largely increased (48, 49). Regardless of the different and potent mechanisms through which these biological drugs with an anti-type 2 inflammation effect modulate the immune system, these drugs were shown to not increase the risk of SARS-CoV-2 infection or worsen the clinical course and outcome of COVID-19 (50). Similarly, in our patients, these drugs did not prevent post-vaccination humoral and cellular immune responses. In addition, the duration of detectable immune responses was comparable to that in a healthy population (14–16). Therefore, the biological drugs used to treat the patients in this study are different from other immunomodulatory drugs, which have been shown to compromise the immunogenicity of the COVID-19 vaccine (51–53).

The COVID-19 vaccine has been shown to elicit SARS-CoV-2-specific immunity in multiple studies, and this capability relates to both humoral and cellular immunity (2–6). The dynamics of the humoral response and its durability in patients in our study were comparable to data obtained from healthy volunteers (54), indicating that the ongoing biological therapy used to treat the study patients had no apparent impact on the dynamics or durability of vaccine-elicited immunity. In agreement with other studies (54), our data also showed that the vaccine promoted stronger CD4^+^ T-cell SARS-CoV-2-specific immunity than CD8^+^ T-cell SARS-CoV-2-specific immunity (33, 55). Additionally, comparable to other reports, the extents of SARS-CoV-2-specific CD4^+^ and CD8^+^ T-cell immunity were correlated (7). In addition, the extent of humoral immunity in our study often correlated with T-cell immunity, namely, with CD8^+^ T-cell immunity. These data, therefore, show that the vaccine proportionally induces different arms of the immune system in severe asthma patients on biological therapy. However, whether this proportionality is skewed by the biological therapy needs to be further investigated.

The immunogenicity of mRNA vaccines decreases with the age of the vaccinated subjects (56, 57). This decrease can be observed in both vaccine-elicited humoral and cellular immunity (56). We also found a correlation between vaccine-elicited humoral immunity and the age of the vaccinated patients after the administration of the first dose of the vaccine and six months after the second dose was administered. These findings indicate that the age of severe asthma patients on biological therapy is a negative predictor of the onset and durability of vaccine-elicited humoral immunity. Surprisingly, patient age did not greatly affect SARS-CoV-2-specific cellular immunity. However, it should be noted that the study did not include patients over the age of 80 years, in whom this decline was found to be most pronounced (56). Regardless, the age of the severe asthma patients in this study played a significant role in the elicited humoral immunity, and similar to considerations for healthy populations, patient age needs to be considered as an indicator for a third booster vaccine dose, as the addition of such a dose was found to significantly restore sufficient SARS-CoV-2-specific immunity (58).

This study showed that patients with severe asthma on biological therapy could achieve activation of SARS-CoV-2-specific immune responses mediated by both arms of the adaptive immune system after a two-dose vaccination regimen with the BNT162b2 mRNA vaccine. However, comparable to those in healthy populations, these immune responses declined and presumably became not protective by 6 months after vaccination, and a booster dose should be considered.

## Data Availability Statement

The raw data supporting the conclusions of this article will be made available by the authors, without undue reservation.

## Ethics Statement

The studies involving human participants were reviewed and approved by The Ethics Committee of the Motol University Hospital in Prague (protocol no. EK-346/21). The patients/participants provided their written informed consent to participate in this study.

## Author Contributions

MP, PT, DmS, and DaS designed the experiments. PT, DmS, MR, JL, and DaS conducted the experiments and/or analyzed the data. MP, AV, PP, and JB supervised the sample collection and clinical aspects of the study. MP and DaS wrote the manuscript. PT, DmS, MR, JL, AV, PP, and JB contributed to the writing of the manuscript. MP and DaS supervised the research. All authors contributed to the article and approved the submitted version.

## Funding

Research in the authors’ laboratories was supported by the institutional IPE2 funding of Charles University, Second Faculty of Medicine in Prague and by funding from the Ministry of Health of the Czech Republic – project NU22-03-00300.

## Conflict of Interest

JB is a part-time employee and a minority shareholder of Sotio, a.s. PP is a member of the advisory board of GSK and received consultancy fees from AstraZeneca, Novartis, and Chiesi and speaker fees from GSK and Novartis.

The remaining authors declare that the research was conducted in the absence of any commercial or financial relationships that could be construed as a potential conflict of interest.

## Publisher’s Note

All claims expressed in this article are solely those of the authors and do not necessarily represent those of their affiliated organizations, or those of the publisher, the editors and the reviewers. Any product that may be evaluated in this article, or claim that may be made by its manufacturer, is not guaranteed or endorsed by the publisher.
