# Supplementary material for: Effectiveness and Durability of mRNA Vaccine-Induced SARS-CoV-2-Specific Humoral and Cellular Immunity in Severe Asthma Patients on Biological Therapy
Source: Front Immunol. 2022 May 20;13:892277. doi: 10.3389/fimmu.2022.892277 (PMC9163958; doi:10.3389/fimmu.2022.892277)
Supplement: Supplementary file 1 [file DataSheet_1.pdf]

SUPPLEMENTARY MATERIAL

Effectiveness and durability of mRNA vaccine-induced SARS-CoV-2-specific humoral and cellular immunity in severe asthma patients on biological therapy

Michal Podrazil[1]*, Pavla Taborska[1]*, Dmitry Stakheev[1]*, Michal Rataj[1], Jan Lastovicka[1], Alena Vlachova[2], Petr Pohunek[3], Jirina Bartunkova[1], Daniel Smrz[1†] (*co-first authors)

Supplementary Table and Figure Legends

Table S1. **Detailed cohort characteristics. ABPA** [allergic bronchopulmonary aspergillosis], **AD** [atopic dermatitis], **A/D** [anxiety/depression], **AR** [allergic rhinoconjunctivitis], **BT** [type of biological therapy; **O** (omalizumab), **M** (mepolizumab), **R** (reslizumab), **B** (benralizumab)], **BTM** [duration of biological therapy in months before vaccination], **C&M D** [cardiovascular & metabolic disease (i.e., diabetes mellitus, hypertension, etc.)], **CRSw/sNP** [chronic rhinosinusitis with/without nasal polyps], **GERD** [gastro-esophageal reflux disease], **Obes** [obesity (BMI≥30)], **OCS** [maintenance oral corticosteroids (mg/day prednisone equivalent)], **OP** [osteoporosis], **OSA** [obstructive sleep apnea], **VCD** [vocal cord dysfunction].

Figure S1. **Serum levels of anti-NCP and anti-RBD antibodies.** (**A**) Serum levels (U/ml) of anti-NCP IgG antibodies before the first vaccine dose (Pre) was administered and 6 months (6 mths) after the second vaccine dose was administered. (**B**) Serum levels (U/ml) of anti-RBD antibodies before the first (Pre) and second (1st) vaccine

doses were administered and 4 weeks (2nd) and 6 months (6 mths) after the second vaccine dose was administered in patients with elevated prevaccination levels of anti-RBD IgA (*left panel*) or IgG (*right panel*) antibodies. (**C**) Serum levels (U/ml) of anti-RBD IgA (*top panels*) or IgG (*bottom panels*) antibodies before the first (Pre) and second (1st) vaccine doses were administered and 4 weeks (2nd) and 6 months (6 mths) after the second vaccine dose was administered in the patients with elevated postvaccination levels of anti-NCP IgG antibodies in **A**. In **A**–**C**, the dotted line indicates the value 18 U/ml. Values above 18 U/ml were considered positive.

Figure S2. **The associations between SARS-CoV-2 spike glycoprotein peptide-reactive CD4$^+$ T cells and the serum levels of anti-RBD antibodies during and after vaccination.** (**A**–**B**) The correlations between the frequencies of peptide-reactive TNFα-, IFNγ-, or TNFα/IFNγ-producing CD4$^+$ T cells and serum levels (U/ml) of anti-RBD IgA (**A**) or IgG (**B**) antibodies before (1st) and 4 weeks (2nd) and 6 months (6 mths) after the second vaccine dose was administered. Spearman's rank-order correlation coefficient (r) and the significance (*p* value; *n* = 34) are indicated.

Figure S3. Demographics and clinical parameters of the biological therapy-stratified groups of patients. The patients were stratified into 3 groups based on the type of biological therapy administered. The groups comprised 17 patients on anti-IgE therapy (O; omalizumab), 13 patients on anti-IL5 therapy (M, mepolizumab), and 3 patients on anti-IL5 (R, reslizumab) therapy. Graphs depicting patient age (*top left*), total IgE serum levels (IU/ml) (*top right*), ECP (ng/ml) (*bottom left*), and blood eosinophil

counts (cells/μl) (*bottom right*) in the stratified groups are shown. Box and whisker plots (2.5–97.5 percentile) are shown, and the significance of differences among the groups (O, M, and R) is indicated ($^{NS}p>0.05$; n = 17 (O), 13 (M), or 3 (R); one-way ANOVA with Dunn's posttest).

Figure S4. **The associations between clinical parameters (total IgE and ECP) and the serum levels of anti-RBD antibodies during and after vaccination.** (**A**) The correlations between total IgE serum levels (IU/ml) and the serum levels (U/ml) of anti-RBD IgA (*top panels*) and IgG (*bottom panels*) antibodies before (1st) and 4 weeks (2nd) and 6 months (6 mths) after the second vaccine dose was administered. (**B**) The correlations between ECP (ng/ml) and the serum levels (U/ml) of anti-RBD IgA (*top panels*) and IgG (*bottom panels*) antibodies before (1st) and 4 weeks (2nd) and 6 months (6 mths) after the second vaccine dose was administered. In **A**–**B**, Spearman's rank-order correlation coefficient (r) and the significance (p value; n = 34) are indicated.

Figure S5. **The associations between total IgE serum levels and SARS-CoV-2 spike glycoprotein peptide-reactive T cells during and after vaccination.** (**A**–**B**) The correlations between total IgE serum levels (IU/ml) and TNFα-, IFNγ-, or TNFα/IFNγ-producing $CD4^+$ (**A**) and $CD8^+$ (**B**) T cells before (1st) and 4 weeks (2nd) and 6 months (6 mths) after the second vaccine dose was administered. In **A**–**B**, Spearman's rank-order correlation coefficient (r) and the significance (p value; n = 34) are indicated.

Figure S6. **The associations between ECP serum levels and SARS-CoV-2 spike glycoprotein peptide-reactive T cells during and after vaccination.** (**A**–**B**) The correlations between ECP (ng/ml) and TNFα-, IFNγ-, or TNFα/IFNγ-producing $CD4^+$ (**A**) and $CD8^+$ (**B**) T cells before (1st) and 4 weeks (2nd) and 6 months (6 mths) after the second vaccine dose was administered. In **A**–**B**, Spearman's rank-order correlation coefficient (r) and the significance (*p* value; *n* = 34) are indicated.

Figure S7. **The associations between the blood eosinophil count and SARS-CoV-2 spike glycoprotein peptide-reactive $CD8^+$ T cells during and after vaccination.** The correlations between the blood eosinophil count (cells/µl) and TNFα-, IFNγ-, or TNFα/IFNγ-producing $CD8^+$ T cells before (1st) and 4 weeks (2nd) and 6 months (6 mths) after the second vaccine dose was administered. Spearman's rank-order correlation coefficient (r) and the significance (*p* value; *n* = 34) are indicated.

Figure S8. **The impact of the blood eosinophil count on the serum levels of anti-RBD antibodies, SARS-CoV-2 spike glycoprotein peptide-reactive $CD4^+$ T cells and patient age during and after the vaccination.** (**A**–**B**) The correlations between the blood eosinophil count (cells/µl) and serum levels (U/ml) of anti-RBD IgA (*top panels*) and IgG (*bottom panels*) antibodies (**A**) or TNFα-, IFNγ-, or TNFα/IFNγ-producing $CD4^+$ T cells (**B**) before (1st) and 4 weeks (2nd) and 6 months (6 mths) after the second vaccine dose was administered. In **A**–**B**, Spearman's rank-order correlation coefficient (r) and the significance (*p* value; *n* = 34) are indicated.

Figure S9. The impact of patient age on SARS-CoV-2 spike glycoprotein peptide-reactive IFNγ- or TNFα/IFNγ-producing T cells during and after vaccination. (**A**) The correlations between patient age and the frequencies of peptide-reactive IFNγ- (*top panels*) or TNFα/IFNγ- (*bottom panels*) producing $CD4^+$ (**A**) and $CD8^+$ (**B**) T cells before (1st) and 4 weeks (2nd) and 6 months (6 mths) after the second vaccine dose was administered. In **A**–**B**, Spearman's rank-order correlation coefficient (r) and the significance (*p* value; *n* = 34) are indicated.

Table S1:

The cohort detailed characteristics

						Asthma comorbidities										
						Upper&lower airways					Extrapulmonary					
**Patient #**	**Gender**	**Age**	**BT**	**BTM**	**OCS**	**AR**	**CRSw/sNP**	**VCD**	**OSA**	**ABPA**	**GERD**	**Obes**	**OP**	**AD**	**A/D**	**C&MD**
1	F	60	M	17	2.5		1				1	1		1		1
2	F	70	M	18			1				1	1				1
3	F	67	O	140	5	1										
4	F	32	M	24	5		1				1		1			1
5	F	45	O	26		1								1		
6	M	70	O	96	5	1							1	1		1
7	F	69	O	88		1					1					
8	M	57	M	10	2.5	1			1		1	1	1	1		1
9	M	48	M	15			1	1			1		1		1	
10	F	26	O	67		1					1		1	1		
11	M	57	O	48		1					1			1		
12	M	73	R	34			1					1	1			
13	M	66	M	44			1			1	1		1			
14	M	69	M	21	5		1									1
15	F	55	M	41			1				1	1				1
16	F	67	M	21			1				1				1	1
17	F	66	O	146		1										
18	F	35	O	40			1									
19	F	46	O	35		1					1			1		
20	F	60	O	85							1	1				1
21*	F	21	O	28		1										
22	M	21	O	98		1										
23	M	47	O	59	5								1	1		1
24*	F	46	R	31			1				1					
25*	M	45	M	38			1									
26	F	62	R	34			1			1					1	
27	M	48	O	78		1						1		1		1
28	F	54	M	30			1	1			1					1
29	M	52	R	30			1				1					1
30	F	51	O	141												
31	F	66	B	9			1		1		1		1			1
32	F	71	O	17		1	1									1
33	M	64	M	7			1				1					1
34	M	58	M	1		1					1					
35	F	36	O	129		1					1					
36	M	58	M	6			1				1					
37	F	45	O	51							1					

*** patients excluded from analyses due to elevated pre-vaccination levels of anti-NCP IgG antibodies in Fig. S1A**

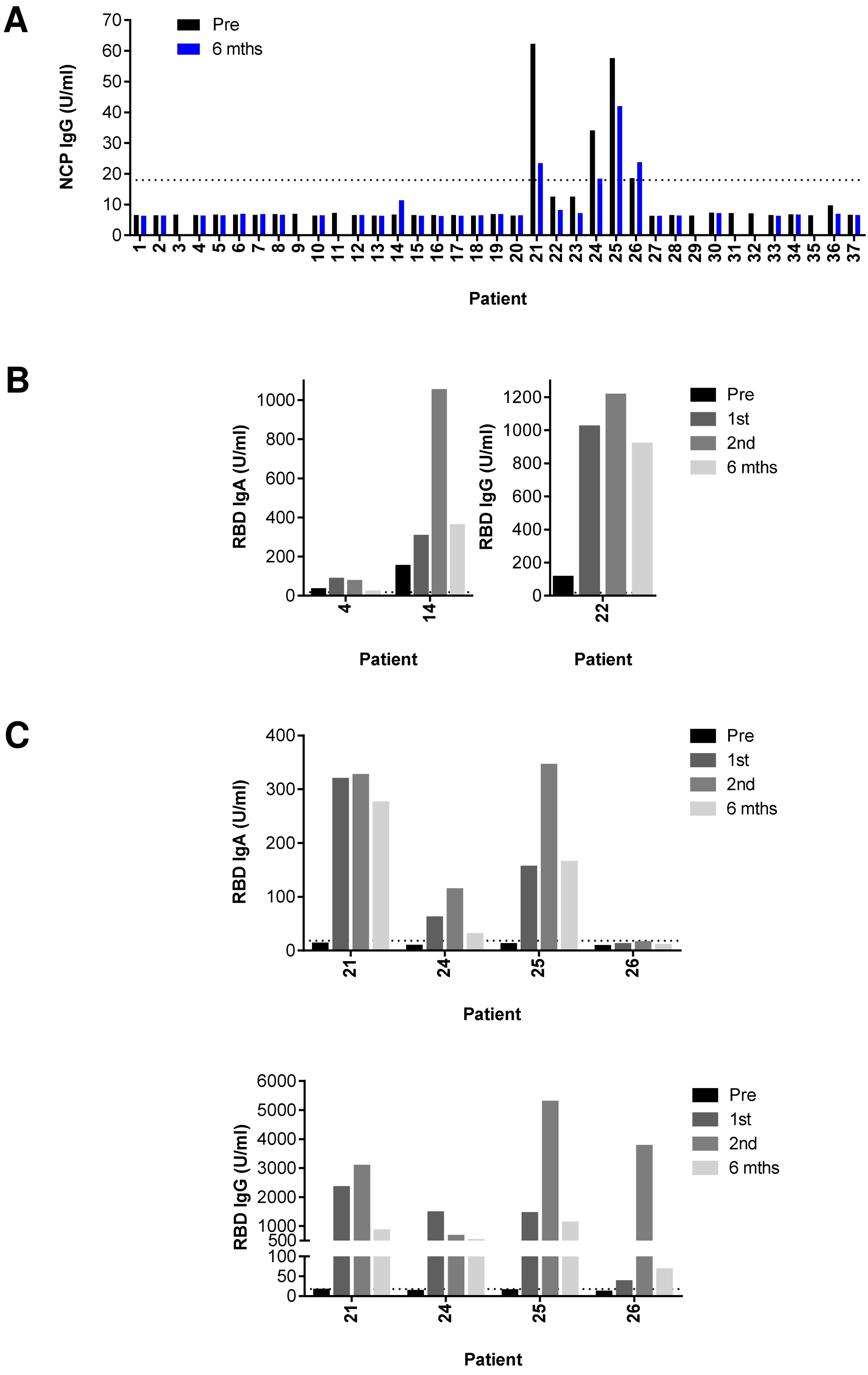

A
Pre
6 mths
NCP IgG (U/ml)
Patient
B
RBD IgA (U/ml)
RBD IgG (U/ml)
Pre
1st
2nd
6 mths
Patient
C
RBD IgA (U/ml)
RBD IgG (U/ml)
Patient

Fig. S1

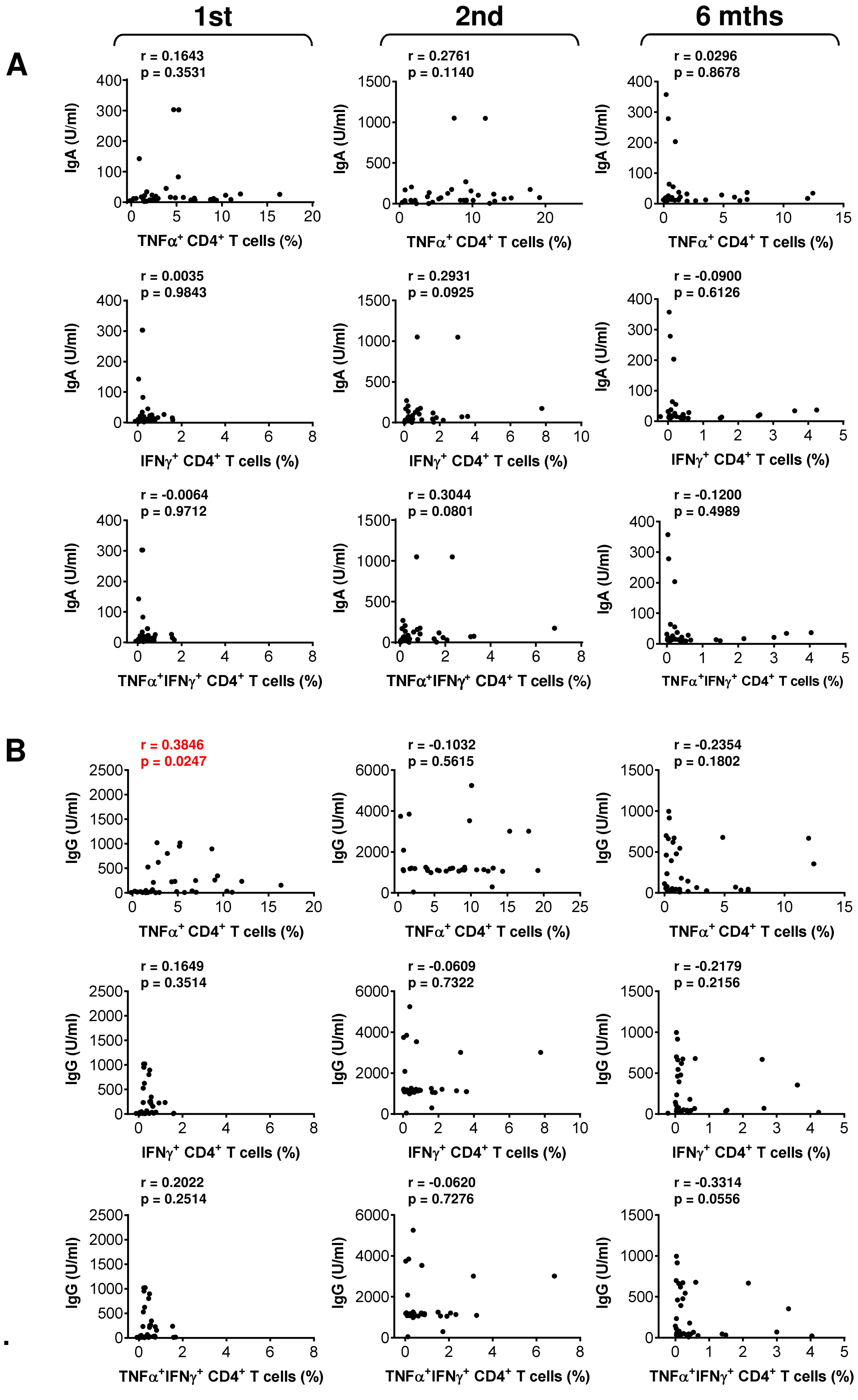
1st
2nd
6 mths
A
r = 0.1643
p = 0.3531
r = 0.2761
p = 0.1140
r = 0.0296
p = 0.8678
IgA (U/ml)
TNFα+ CD4+ T cells (%)
r = 0.0035
p = 0.9843
r = 0.2931
p = 0.0925
r = -0.0900
p = 0.6126
IFNγ+ CD4+ T cells (%)
r = -0.0064
p = 0.9712
r = 0.3044
p = 0.0801
r = -0.1200
p = 0.4989
TNFα+IFNγ+ CD4+ T cells (%)
B
r = 0.3846
p = 0.0247
r = -0.1032
p = 0.5615
r = -0.2354
p = 0.1802
IgG (U/ml)
TNFα+ CD4+ T cells (%)
r = 0.1649
p = 0.3514
r = -0.0609
p = 0.7322
r = -0.2179
p = 0.2156
IFNγ+ CD4+ T cells (%)
r = 0.2022
p = 0.2514
r = -0.0620
p = 0.7276
r = -0.3314
p = 0.0556
TNFα+IFNγ+ CD4+ T cells (%)

Fig. S2

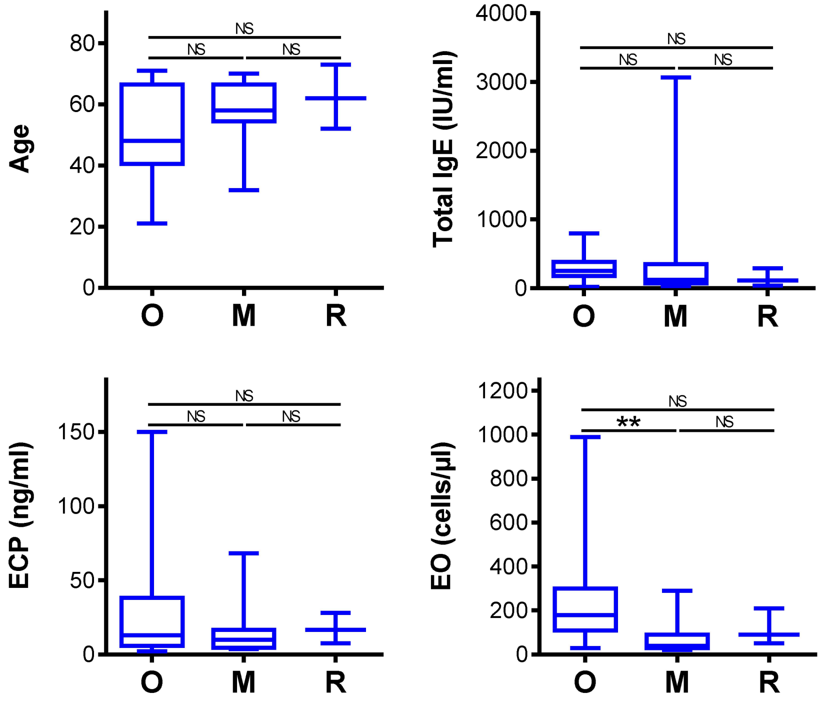
Age
0
20
40
60
80
NS
NS
NS
O
M
R
Total IgE (IU/ml)
0
1000
2000
3000
4000
NS
NS
NS
O
M
R
ECP (ng/ml)
0
50
100
150
NS
NS
NS
O
M
R
EO (cells/µl)
0
200
400
600
800
1000
1200
NS
**
NS
O
M
R

Fig. S3

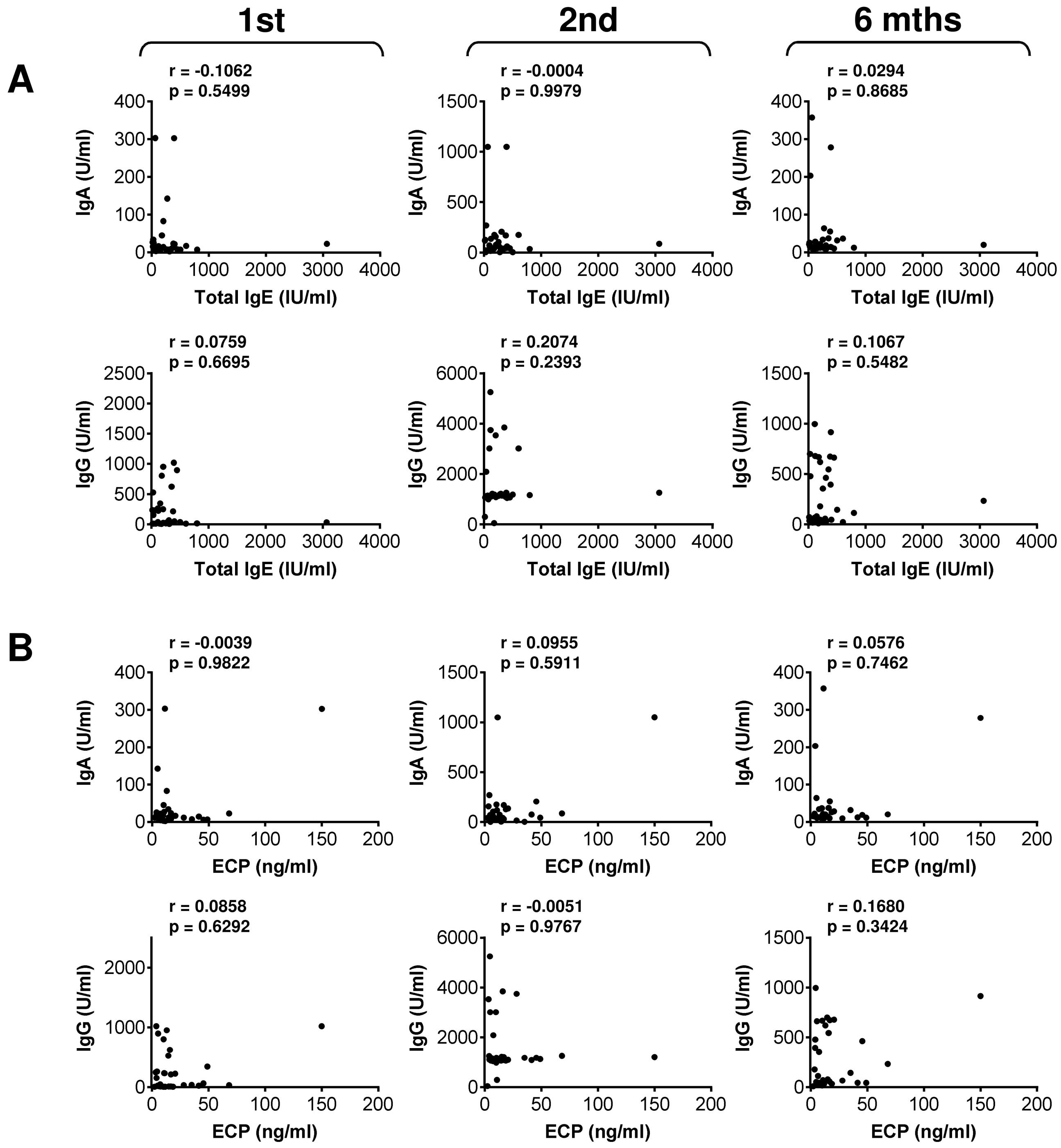
1st
2nd
6 mths
A
r = -0.1062
p = 0.5499
r = -0.0004
p = 0.9979
r = 0.0294
p = 0.8685
IgA (U/ml)
Total IgE (IU/ml)
r = 0.0759
p = 0.6695
r = 0.2074
p = 0.2393
r = 0.1067
p = 0.5482
IgG (U/ml)
Total IgE (IU/ml)
B
r = -0.0039
p = 0.9822
r = 0.0955
p = 0.5911
r = 0.0576
p = 0.7462
IgA (U/ml)
ECP (ng/ml)
r = 0.0858
p = 0.6292
r = -0.0051
p = 0.9767
r = 0.1680
p = 0.3424
IgG (U/ml)
ECP (ng/ml)

Fig.
S4

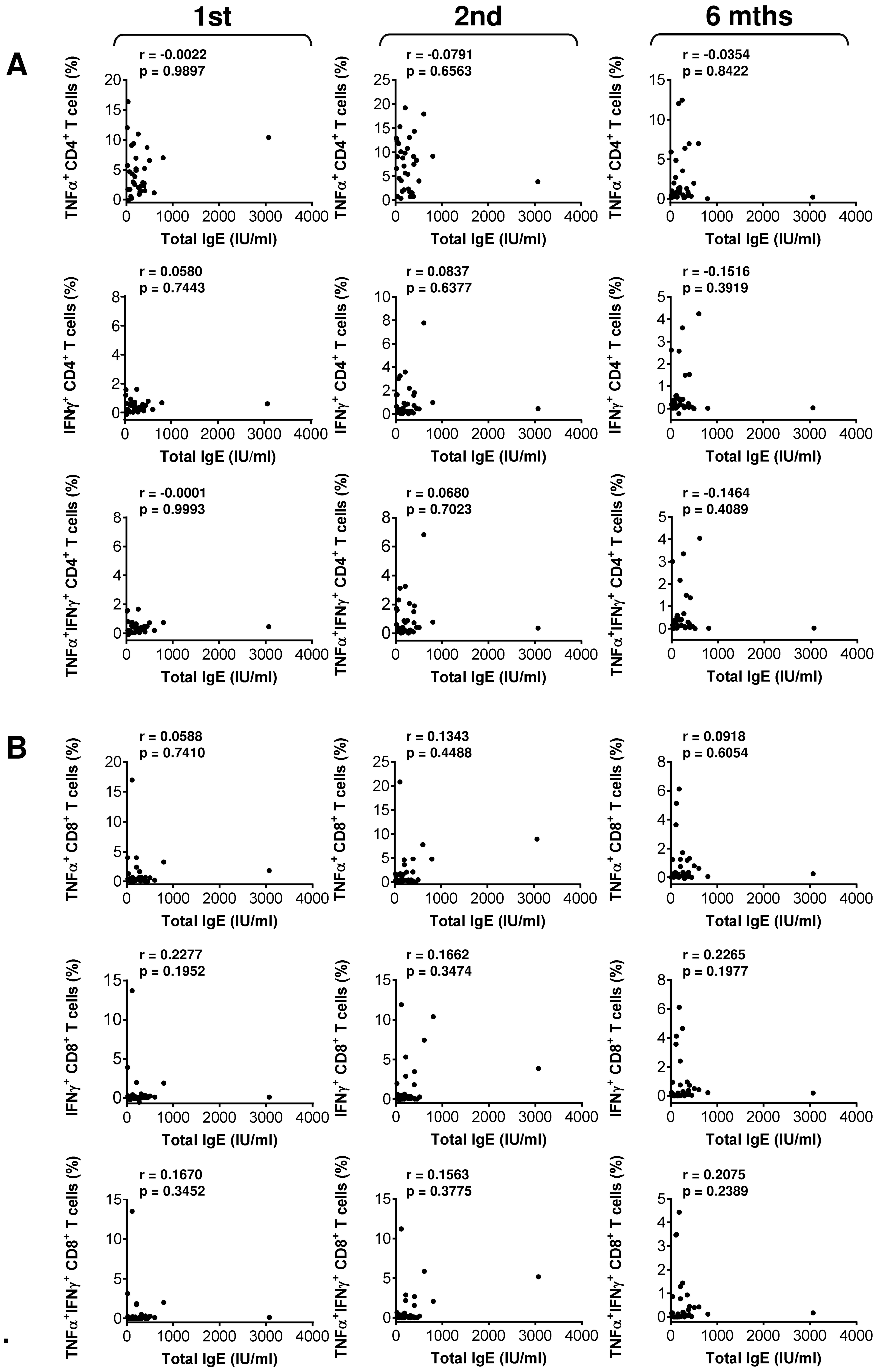

1st
2nd
6 mths
A
r = -0.0022
p = 0.9897
r = -0.0791
p = 0.6563
r = -0.0354
p = 0.8422
r = 0.0580
p = 0.7443
r = 0.0837
p = 0.6377
r = -0.1516
p = 0.3919
r = -0.0001
p = 0.9993
r = 0.0680
p = 0.7023
r = -0.1464
p = 0.4089
B
r = 0.0588
p = 0.7410
r = 0.1343
p = 0.4488
r = 0.0918
p = 0.6054
r = 0.2277
p = 0.1952
r = 0.1662
p = 0.3474
r = 0.2265
p = 0.1977
r = 0.1670
p = 0.3452
r = 0.1563
p = 0.3775
r = 0.2075
p = 0.2389
TNFα+ CD4+ T cells (%)
IFNγ+ CD4+ T cells (%)
TNFα+IFNγ+ CD4+ T cells (%)
TNFα+ CD8+ T cells (%)
IFNγ+ CD8+ T cells (%)
TNFα+IFNγ+ CD8+ T cells (%)
Total IgE (IU/ml)

Fig. S5

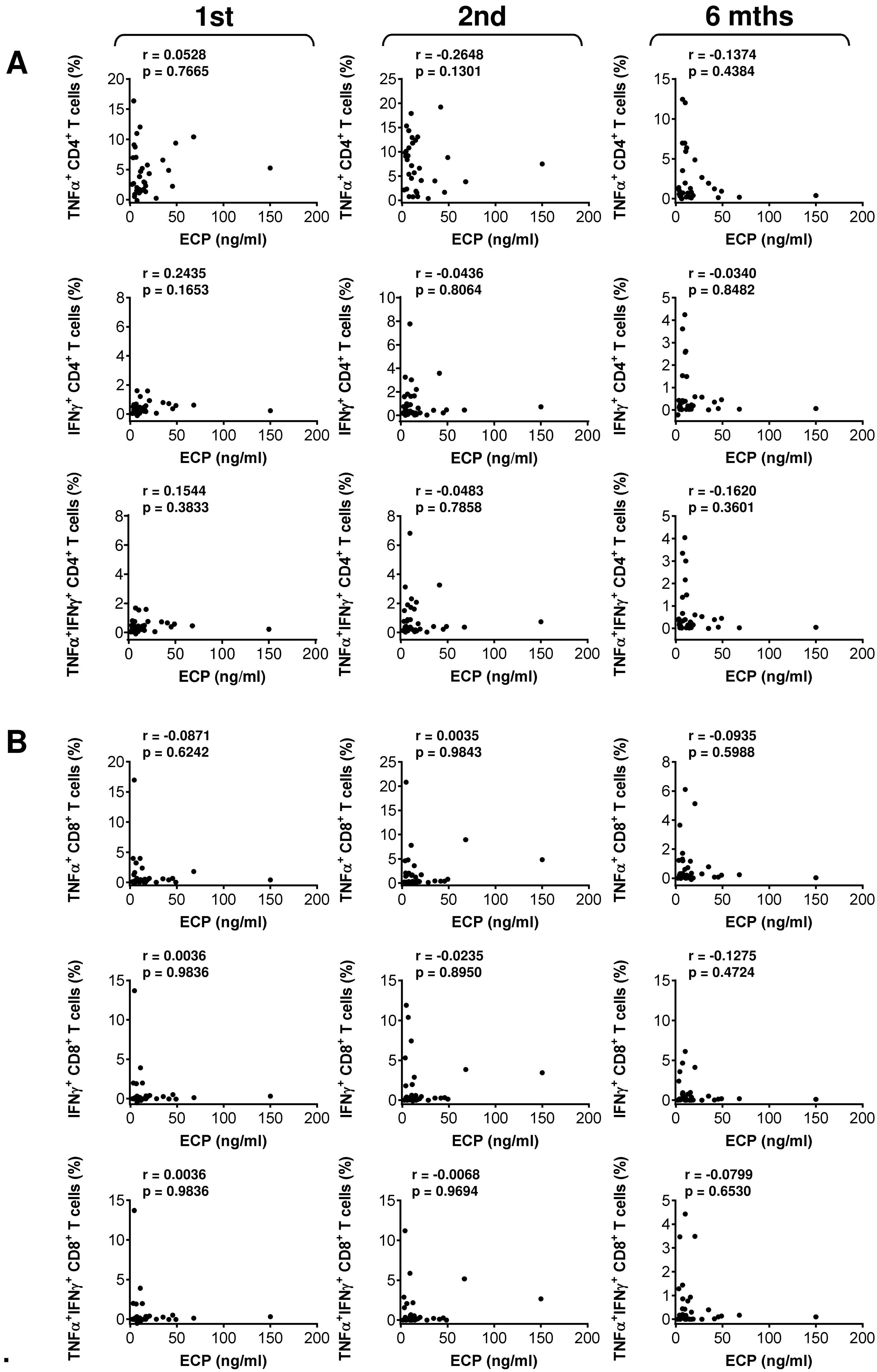

1st
2nd
6 mths
A
B
r = 0.0528
p = 0.7665
r = -0.2648
p = 0.1301
r = -0.1374
p = 0.4384
r = 0.2435
p = 0.1653
r = -0.0436
p = 0.8064
r = -0.0340
p = 0.8482
r = 0.1544
p = 0.3833
r = -0.0483
p = 0.7858
r = -0.1620
p = 0.3601
r = -0.0871
p = 0.6242
r = 0.0035
p = 0.9843
r = -0.0935
p = 0.5988
r = 0.0036
p = 0.9836
r = -0.0235
p = 0.8950
r = -0.1275
p = 0.4724
r = 0.0036
p = 0.9836
r = -0.0068
p = 0.9694
r = -0.0799
p = 0.6530
TNFα+ CD4+ T cells (%)
IFNγ+ CD4+ T cells (%)
TNFα+IFNγ+ CD4+ T cells (%)
TNFα+ CD8+ T cells (%)
IFNγ+ CD8+ T cells (%)
TNFα+IFNγ+ CD8+ T cells (%)
ECP (ng/ml)

Fig.
S6

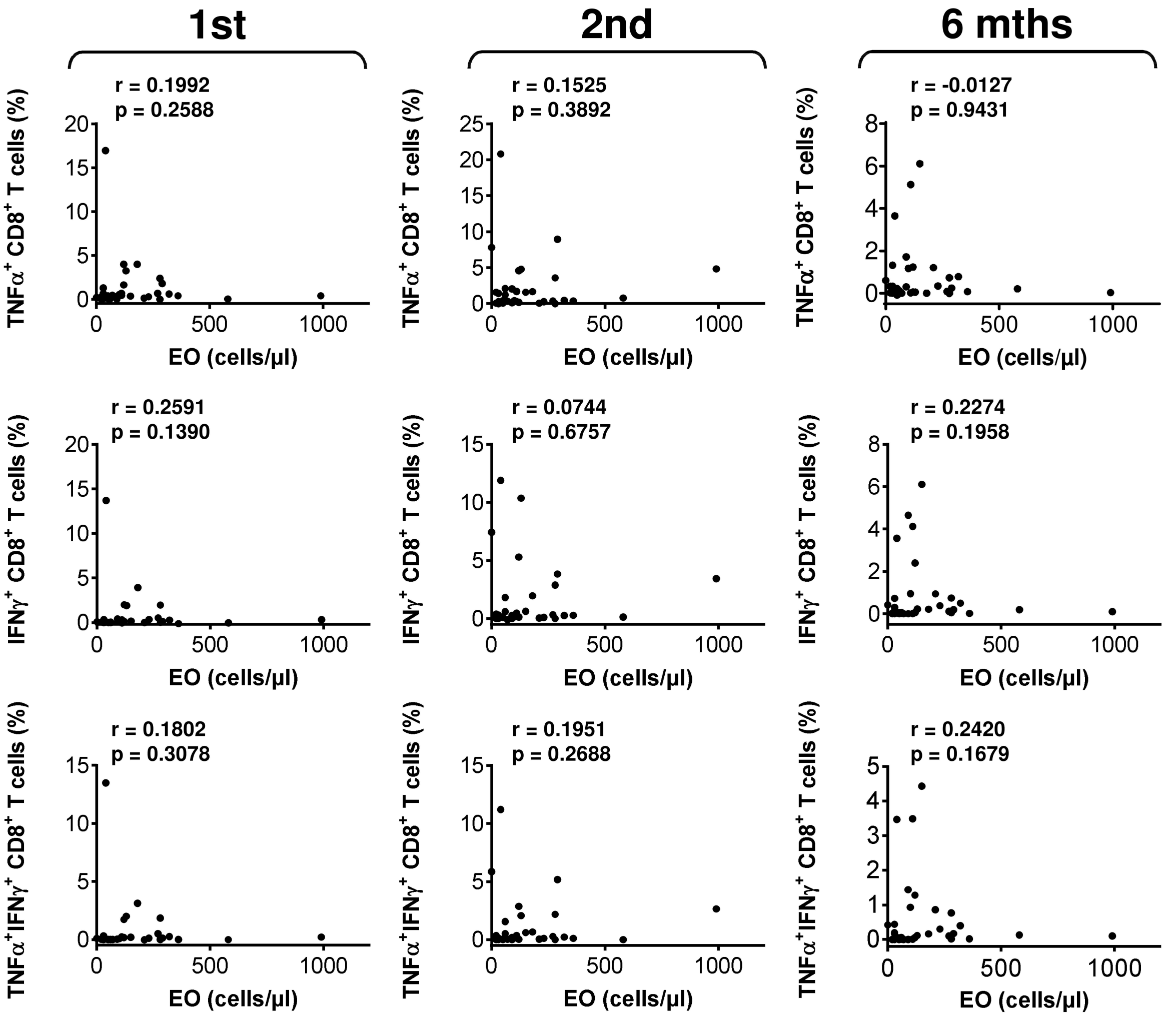
1st
2nd
6 mths
r = 0.1992
p = 0.2588
r = 0.1525
p = 0.3892
r = -0.0127
p = 0.9431
TNFα+ CD8+ T cells (%)
EO (cells/µl)
r = 0.2591
p = 0.1390
r = 0.0744
p = 0.6757
r = 0.2274
p = 0.1958
IFNγ+ CD8+ T cells (%)
EO (cells/µl)
r = 0.1802
p = 0.3078
r = 0.1951
p = 0.2688
r = 0.2420
p = 0.1679
TNFα+IFNγ+ CD8+ T cells (%)
EO (cells/µl)

Fig.
S7

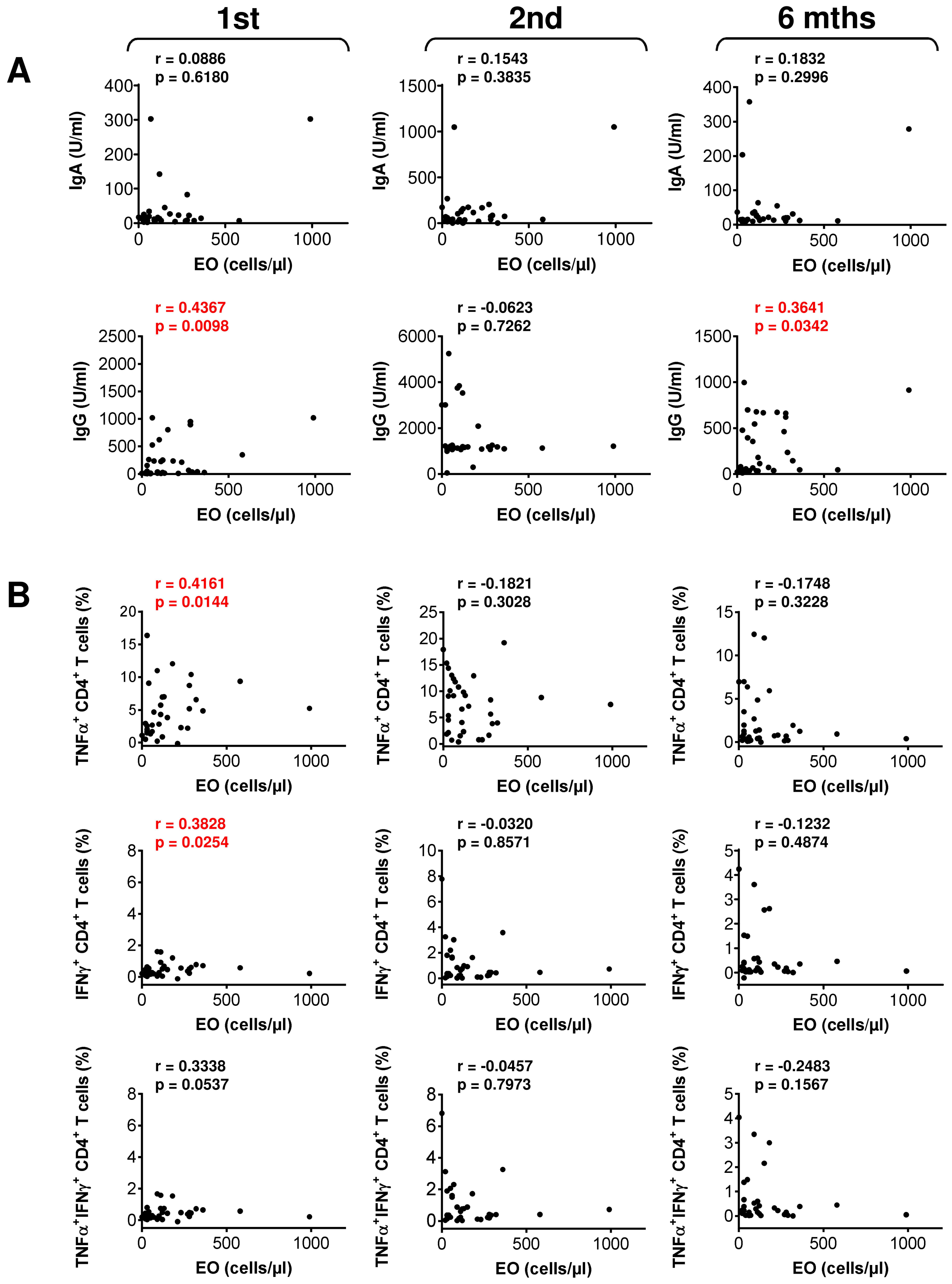

1st
2nd
6 mths
A
r = 0.0886
p = 0.6180
r = 0.1543
p = 0.3835
r = 0.1832
p = 0.2996
IgA (U/ml)
EO (cells/µl)
r = 0.4367
p = 0.0098
r = -0.0623
p = 0.7262
r = 0.3641
p = 0.0342
IgG (U/ml)
B
r = 0.4161
p = 0.0144
r = -0.1821
p = 0.3028
r = -0.1748
p = 0.3228
TNFα+ CD4+ T cells (%)
r = 0.3828
p = 0.0254
r = -0.0320
p = 0.8571
r = -0.1232
p = 0.4874
IFNγ+ CD4+ T cells (%)
r = 0.3338
p = 0.0537
r = -0.0457
p = 0.7973
r = -0.2483
p = 0.1567
TNFα+IFNγ+ CD4+ T cells (%)

Fig. S8

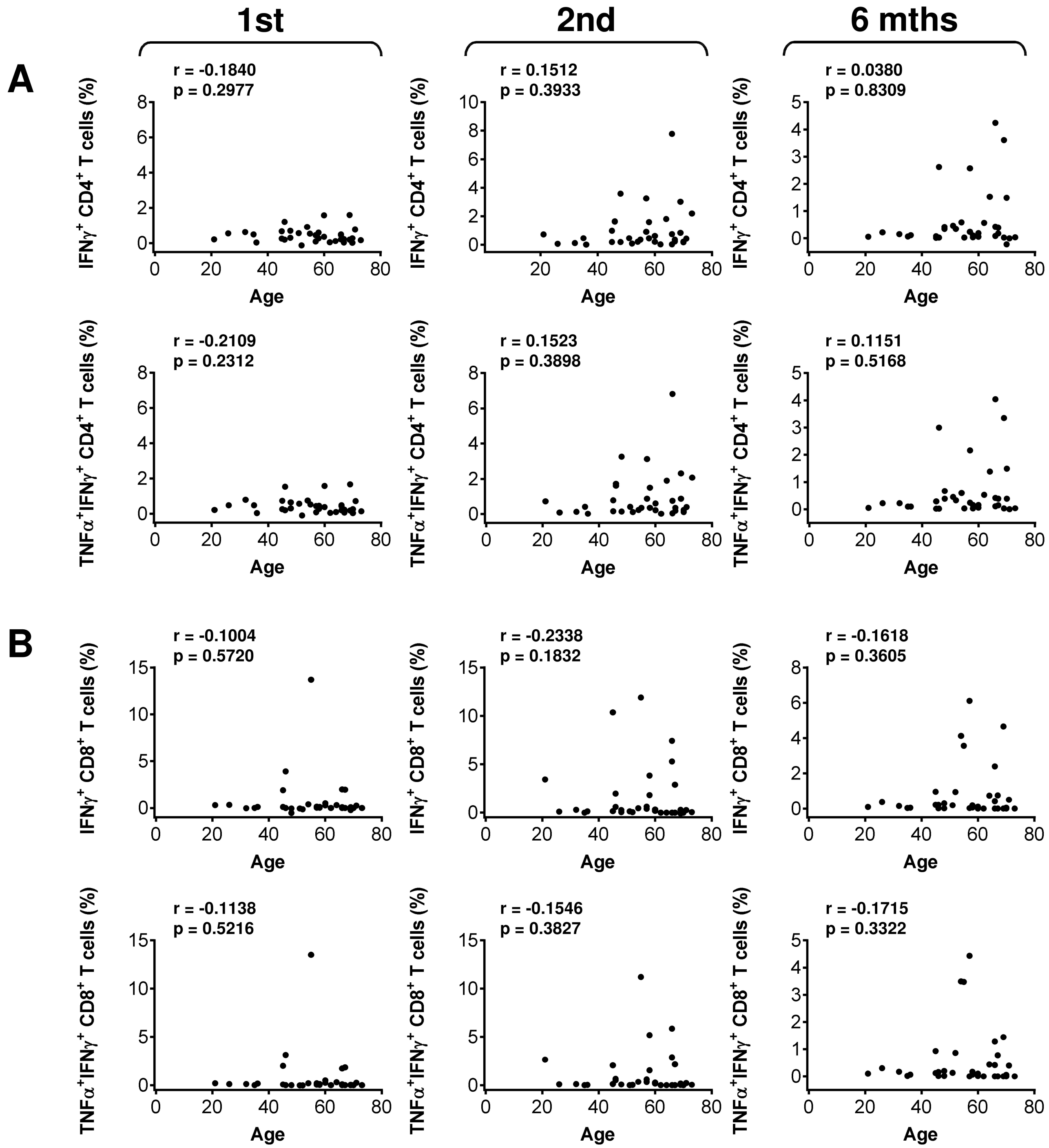
1st
2nd
6 mths
A
r = -0.1840
p = 0.2977
r = 0.1512
p = 0.3933
r = 0.0380
p = 0.8309
IFNγ+ CD4+ T cells (%)
Age
r = -0.2109
p = 0.2312
r = 0.1523
p = 0.3898
r = 0.1151
p = 0.5168
TNFα+IFNγ+ CD4+ T cells (%)
B
r = -0.1004
p = 0.5720
r = -0.2338
p = 0.1832
r = -0.1618
p = 0.3605
IFNγ+ CD8+ T cells (%)
r = -0.1138
p = 0.5216
r = -0.1546
p = 0.3827
r = -0.1715
p = 0.3322
TNFα+IFNγ+ CD8+ T cells (%)

Fig.
S9
